# Societies in Motion: Understanding Age Differences in the Perceived Impact of Societal Transitions

**DOI:** 10.1093/geroni/igaf122.2282

**Published:** 2025-12-31

**Authors:** Patrick Klaiber, John Bechara, Loes Abrahams, Bell Piyasinchai, Tom Junker, Meeke Hoedjes, Stefan Bogaerts

**Affiliations:** Tilburg University, Tilburg, Noord-Brabant, Netherlands; Tilburg University, Tilburg, Noord-Brabant, Netherlands; Tilburg University, Tilburg, Noord-Brabant, Netherlands; Tilburg University, Tilburg, Noord-Brabant, Netherlands; Tilburg University, Tilburg, Noord-Brabant, Netherlands; Tilburg University, Tilburg, Noord-Brabant, Netherlands; Tilburg University, Tilburg, Noord-Brabant, Netherlands

## Abstract

Societal transitions such as climate change and digitalization are reshaping daily life, yet individuals differ in how they experience their impact. Despite growing research on these changes, little is known about age differences in their perceived impact. Using data from the Tilburg University Monitor of Social and Behavioral Issues, a representative sample of the Dutch population, we analyzed responses from 4,891 participants aged 16-96 (M = 54.75, 52% female) who completed an online questionnaire in December 2024. Participants rated the perceived impact of six societal transitions: (1) climate change and sustainability, (2) digitalization, (3) diversity and inclusion, (4) demographic changes, (5) international conflicts, and (6) lack of living space. Structural equation modeling was used to examine age as a predictor of both a generalized sense of impact (common factor) and the specific impact of each transition. Our findings showed clear age-related differences in the perception of societal changes. Older adults generally perceived societal transitions as less impactful than younger adults. However, when adjusting for this general trend, older adults felt more affected by climate change, demographic shifts, and international conflicts, while they felt less impacted by digitalization, diversity and inclusion, and housing shortages. These findings suggest that while older adults may feel generally more detached from societal change, they do recognize the influence of transitions that pose broad, existential challenges. This study provides first insights into generational differences in the experience of societal change, with important implications for designing inclusive policies that address the concerns of all age groups.

